# Multiple system atrophy: Diagnostic challenges and a proposed diagnostic algorithm

**DOI:** 10.1016/j.prdoa.2024.100271

**Published:** 2024-09-18

**Authors:** Deepmala Nandanwar, Daniel D. Truong

**Affiliations:** The Parkinson and Movement Disorder Institute, 9940 Talbert Avenue, Fountain Valley, CA 92708, USA

## Abstract

Multiple system atrophy (MSA) is a heterogenous condition, presenting with core clinical features of autonomic dysfunction, parkinsonism, and/or cerebellar ataxia. The presence of alpha-synuclein glial cytoplasmic inclusion is the hallmark of MSA. It shares a common pathological origin with Parkinson’s disease (PD) and Lewy body dementia (DLB) and they are collectively grouped as “synucleinopathies.” The pathological synuclein protein is now well- recognized in skin biopsies of these patients. Besides the pathological findings, radiological investigation is a useful diagnostic tool. Brain MRI helps rule out other etiologies, and findings like the “Hot-cross bun” sign, “putaminal atrophy,” and “infratentorial findings” can assist with the diagnosis of MSA. Cardiac MIBG scan, autonomic testing, urodynamic studies can help differentiate MSA from other conditions. Although diagnostic tools are available for MSA diagnosis, clarity is needed on when to use these tests. We suggest a diagnostic algorithm to navigate the use of these tests. However, this algorithm is not intended to replace the use of current MDS diagnostic criteria of MSA.

## 1 Introduction

Multiple system atrophy is a unique form of atypical parkinsonism that has diverse clinical presentation. The first case of olivopontocerebellar degeneration was described by Dejerine and Thomas in 1900 [1]. Later, Shy and Drager in 1960 described the neuropathological features in cases with neurological symptoms and orthostatic hypotension [2]. Due to the heterogenous presentation of the disease, Oppenheimer in 1969 renamed this entity as multiple system atrophy (MSA) [3]. Today, MSA is clinically described as a condition with autonomic dysfunction presenting with parkinsonism (MSA-P) and/or cerebellar features (MSA-C).

The prevalence of MSA ranges from 1.9 to 4.9 per 100,000 with the incidence of 0.1 to 3 per 100,000 [4]. MSA-P predominates in North American and European populations, whereas MSA-C is more prevalent among Japanese patients [5]. The estimated median survival from the time of symptom onset is about 9.8 years [6]. The diagnosis of MSA can be challenging and requires comprehensive evaluation. Unfortunately, delays in diagnosis and misdiagnoses are common. A timely diagnosis is important to enable patients and caregivers to plan their future; physicians can offer appropriate counseling and treatment to the patient with the correct diagnosis. The aim of this article is to discuss the diagnostic challenges, followed by the discussion of current MSA clinical criteria suggested by MDS (Movement Disorder Society) and the optimal use of available diagnostic tools to guide the MSA diagnosis.

## 2 Diagnostic criteria and challenges

Quinn was first to propose the diagnostic criteria for MSA in 1989, classifying it as SND (Striatonigral degeneration; Predominant Parkinson’s type) and OPCA (olivopontocerebellar atrophy; predominantly cerebellar type) and proposing that cases be further described as “possible,” “probable,” or “definite” based on clinical and pathological findings [7]. The knowledge of glial cytoplasmic inclusions (GCIs) in oligodendrocytes of MSA patients was provided by Papp in 1989 [8] and the first consensus statement in 1998 used the presence of GCI along with degeneration of straitonigral and olivopontocerebellar pathways for pathological confirmation of MSA. They continued the use of “possible,” “probable,” and “definite” diagnostic descriptors MSA based on clinicopathological assessment; however, they discarded the use of the SND and OPCA classifications. Instead, the consensus recommended that cases be classified as either MSA-P for Parkinson’s predominant and MSA-C for cerebellar predominant MSA [9].

Osaki and colleagues used samples from the Queen Square Brain Bank to evaluate the validity of these early criteria. They reported both the Quinn and first consensus statement diagnostic criteria improved clinical diagnosis of MSA early in the disease course, yet neither improved accuracy in later stage of disease. The consensus diagnostic criteria exhibited high positive predictive value (PPV 91 %) but low sensitivity (63 %) for diagnosis of probable MSA compared to the diagnosis of possible MSA (PPV 86 %, sensitivity 92 %) during last clinic visit [10].

Additional research continued to refine diagnosis of MSA. Subsequently in 2008, a second consensus document on MSA diagnostic criteria was published, which added the presence of GCI formed by fibrillary alpha- synuclein protein, along with degeneration of striatonigral and olivopontocerebellar structures for pathological confirmation of MSA. They also suggested the use of additional radiological findings like MRI-brain,18F-fluoro-2-deoxy-D-glucose positron emission tomography (FDG-PET), single photon emission CT (SPECT) to classify possible MSA [11].

Osaki and colleagues reapplied the 2008 consensus criteria to the original postmortem samples from their initial study. Their findings revealed that for possible MSA cases, the 2008 criteria demonstrated the improved sensitivity and PPV during first clinic visit (Sensitivity and PPV; 41 % and 95 % vs 28 % and 93 %), but same sensitivity and improved PPV for last clinic visit, compared to 1998 criteria (Sensitivity and PPV; 92 % and 89 % vs 92 % and 86 %) [12].

Finally, in 2019, the MDS MSA Study Group critically analyzed the low sensitivity and specificity of the 2008 consensus MSA criteria [13], leading to proposal of 2022 MDS criteria. The new criteria introduce “clinically probable,” and “clinically established” diagnostic categories. They emphasis on importance of presence of essential features, motor and non-motor features, radiological features as well as exclusion criteria. The introduction of possible prodromal MSA was suggested for research purposes (Table 1) [14].

**Table 1:** Movement disorder society criteria for diagnosis of Multiple system atrophy, adapted and modified [14].

**Essential feature**
Sporadic and progressive disease in adult >30 years age
**Core clinic features**
*For clinically established MSA*
A. Autonomic dysfunction (one of the following)
1. Unexplained urinary voiding difficulties with > 100 cc post void residual
2. Unexplained urge in continence
3. Neurogenic orthostatic hypotension = Drop of SBP≥20 mmhg or DBP≥10 mmhg within 3 mins of standing or head tilt test
B. L-dopa poorly responsive parkinsonism
C. Cerebellar ataxia (two features of gait ataxia, limb ataxia, oculomotor dysfunction, cerebellar dysarthria)
*For clinically probable MSA*
A. Autonomic dysfunction (one of the following)
1. Unexplained urinary voiding difficulties with post void residual
2. Unexplained urge in continence
3. Neurogenic orthostatic hypotension = Drop of SBP≥20 mmhg or DBP≥10 mmhg within 10 mins of standing or head tilt test
B. Parkinsonism
C. Cerebellar ataxia (one features of gait ataxia, limb ataxia, oculomotor dysfunction, cerebellar dysarthria
**Supportive clinical features**
A. Motor features
1. Rapid progression within 3 years of motor symptom onset
2. Early onset balance problem (moderate to severe) within 3 years of symptom onset
3. Severe dysarthria within 3 years of motor symptom onset
4. Severe dysphagia within 3 years of motor symptom onset
5. Unexplained Babinski sign
6. Craniofacial dystonia which may get worse with L-dopa therapy and in absence of limb dyskinesia
7. Postural deformity
8. Jerky myoclonus quality to postural or kinetic tremor
B. Non-motor feature
1. Stridor
2. Inspiratory sigh
3. Erectile dysfunction in < 60 years of age with clinically probable MSA (but not isolated erectile dysfunction)
4. Pathological laughter and crying
5. Cold and discolored hands and feet
**MRI marker**
*For MSA-P*
1. Atrophy of putamen (and decrease signal on iron- sensitive sequence), middle cerebellar peduncle (MCP), pons, cerebellum
2. Increase putamen, MCP diffusivity
3. “Hot cross bun” sign
*For MSA-C*
1. Atrophy of putamen (and decrease signal on iron- sensitive sequence), infratentorial structures (middle cerebellar peduncle (MCP), pons)
2. Increase putamen diffusivity
3. “Hot cross bun” sign
**Exclusion criteria**
Persistent beneficial response to dopaminergic therapy
Dementia (DSM-V) within 3 years of motor disease onset
Vertical Gaze palsy or slow vertical gaze
Unexplained anosmia
Hallucination unrelated to medications within 3 years of disease onset
Fluctuation in the alertness, cognition and early decline in visuosperceptual abilities
MRI brain suggestive for alternative diagnosis (e.g. Multiple sclerosis, PSP, vascular PD etc.)
Alternative diagnosis to explain patient’s symptoms of parkinsonism, ataxia, autonomic dysfunction

**Clinically established MSA**
1. Essential feature
2. Core clinical feature
a. One feature of autonomic dysfunction and
b. L-dopa poorly responsive parkinsonism OR Cerebellar ataxia
3. Two supportive clinical features
4. At least one MRI marker
5. Absence of exclusion criteria
**Clinically probable MSA**
1. Essential feature
2. Core clinical feature
a. One feature of autonomic dysfunction OR
b. L-dopa poorly responsive parkinsonism OR
c. Cerebellar ataxia
3. One supportive clinical feature
4. Absence of exclusion criteria

Sekiya and colleagues had conducted a retrospective analysis on patients with clinically or pathologically MSA diagnosis from Mayo clinic Brain Bank to evaluate the validity of MDS MSA criteria. They concluded 99 % specificity and 16 % sensitivity for clinically established MSA and 74 % specificity and 64 % sensitivity in clinically probable MSA patients [15].

## 3 Diagnostic considerations

The diagnosis of MSA should be considered in individuals aged 30 and above who present with a sporadic onset and progressive deterioration of neurological function, characterized by a combination of autonomic dysfunction, parkinsonism, and/or cerebellar ataxia. It is noteworthy that autonomic dysfunction may manifest years before the appearance of cerebellar or parkinsonian symptoms. Additionally, features such as early postural instability, dysarthria, dysphagia within three years of motor symptom onset, unexplained Babinski sign, jerky tremor, camptocormia, anterocollis, Pisa syndrome, contractures of the hands and feet, stridor, inspiratory sigh, pseudobulbar affect, polysomnography-proven REM sleep behavior disorder, and cold extremities should be carefully evaluated and documented, as they support the diagnosis of MSA. The heterogeneous and variable nature of presentations present challenges in diagnosing MSA accurately. It is crucial to implement the current MDS criteria as accurately as possible to establish the core clinical features (for detail, please refer to Table 1). While the MDS criteria provides clinical framework for the MSA diagnosis, situations may arise where diagnosis remains difficult. These challenges may emerge when a patient is unable to provide a clear medical history or presents with ambiguous clinical features.

In these situations, we recommend adopting a systematic approach (Fig. 2, Fig. 3) that begins with a clinical suspicion of MSA. It is important to note that the tests mentioned below should be used with caution, as they lack robust clinical data to support their use without the appropriate clinical context. Another limitation of these tests is their limited availability in general practice.

**Fig. 1:**
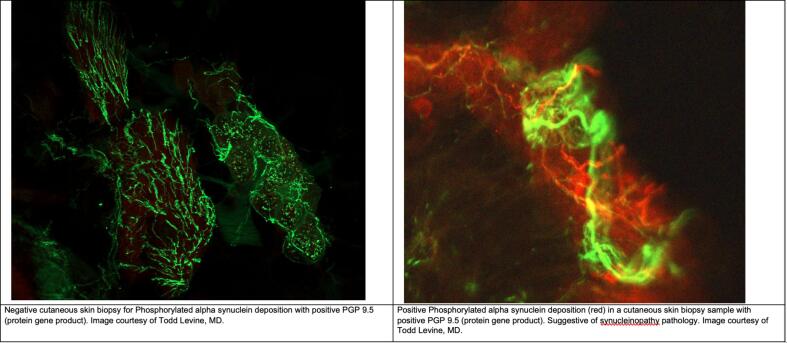
Examples of negative cutaneous skin biopsy for Phosphorylated alpha synuclein deposition (left panel) and positive Phosphorylated alpha synuclein deposition (red) in a cutaneous skin biopsy sample (right panel).

**Fig. 2:**
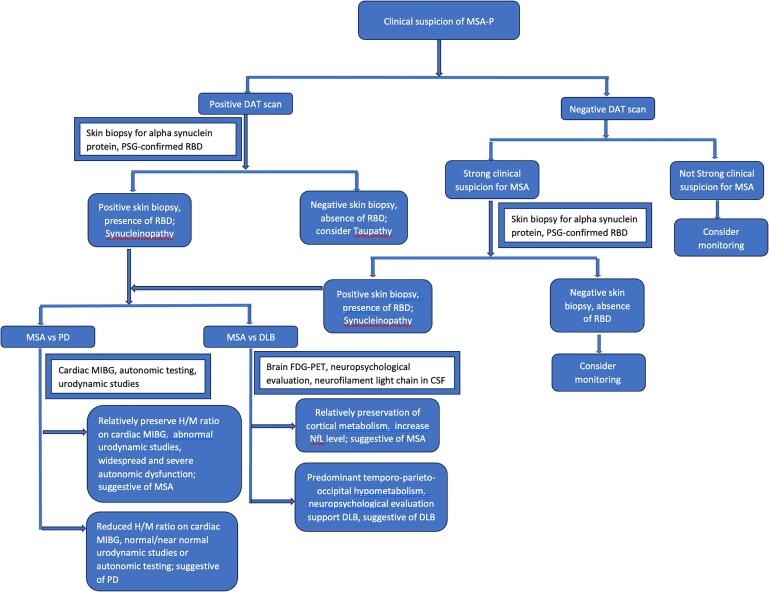
A systematic approach for optimal utilization of diagnostic tools for diagnosing MSA-P. PSG: polysomnography; RBD: REM sleep behavior disorder; H/M ratio: Heart/mediastenium ratio; PD: Parkinson’s disease; DLB: Lewy body dementia; MSA-P: Multiple system atrophy-parkinson’s type; Cardiac MIBG scan: Cardiac Metaiodobenzylguanidine scan; FDG-PET: 18F-fluoro-2-deoxy-D-glucose positron emission tomography; DAT scan: Dopamine transporter SPECT scan.

**Fig. 3:**
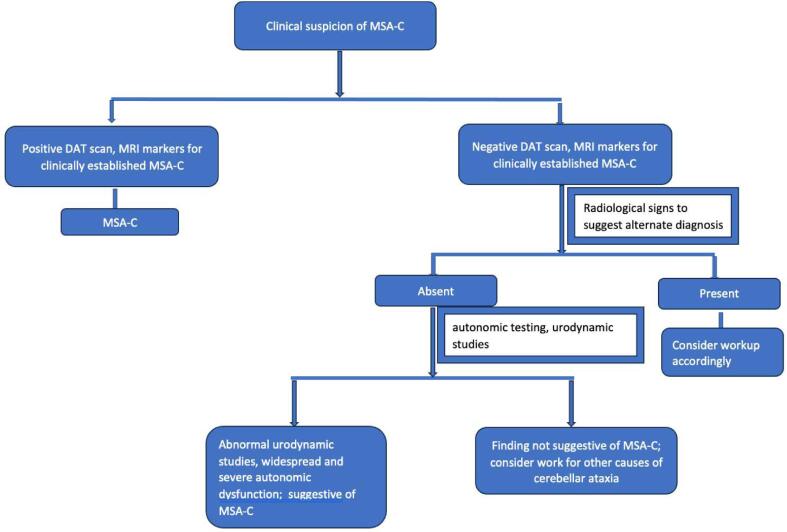
A systematic approach for optimal utilization of diagnostic tools for diagnosing MSA-C, DAT scan: Dopamine transporter SPECT scan; MSA-C: Multiple system atrophy-cerebellar type, DAT scan: Dopamine transporter SPECT scan; MSA-C: Multiple system atrophy-cerebellar type.

Initial work up

Patients should undergo appropriate laboratory work-up based on their clinical presentation. In cases of dysautonomia, conditions such as diabetes, amyloidosis, pure autonomic failure, or paraneoplastic etiologies (e.g., anti-Hu antibody, anti-CV2/CRMP5 associated neuropathy) should be considered. Similarly, in cases of cerebellar ataxia, other potential causes such as alcohol-related ataxia, medication-induced ataxia (e.g., certain anti-epileptics, chemotherapeutic agents, amiodarone), vitamin B1 and E deficiencies, heavy metal toxicity, thyroid dysfunction, rheumatological conditions, paraneoplastic conditions (e.g., anti-Hu, anti-Ri, anti-CV2/CRMP5 antibodies), immune-mediated etiologies, genetic etiologies (e.g., Fragile X-associated tremor/ataxia syndrome [FXTAS], spinocerebellar ataxias, Friedreich's ataxia), and sporadic adult-onset ataxia should be considered [16]. All patients should undergo a brain scan, preferably MRI-brain, along with other necessary lab tests. The current MDS criteria for MSA emphasize the utility of MRI markers, including atrophy of putamen, middle cerebellar peduncle (MCP), pons and cerebellum; increase diffusivity of putamen, MCP and “Hot cross bun” sign. Even a normal MRI can be a marker for clinically probable MSA. The increase diffusivity of putamen has shown to differentiate MSA from PD with a sensitivity of 77 %-100 % and specificity of 80 %-100 %[17]. The MRI brain imaging not only useful for supporting MSA diagnosis but this may also assist in ruling out other diagnosis that may mimic MSA like PSP (progressive supranuclear palsy), vascular parkinsonism, multiple sclerosis or other etiology of cerebellar ataxia. While the “Hot cross bun” sign, MCP sign has been shown to have excellent specificity with MSA diagnosis but they lack good sensitivity [17]. Therefore, MRI-brain should not be used to exclude diagnosis of MSA. The diffusion-weighted MRI sequence is also included in the current MDS-MSA criteria for the diagnosis of clinically established MSA. Increased diffusivity of the putamen and MCP is suggested for the diagnosis of clinically established MSA-P, while increased diffusivity of the putamen is suggested for clinically established MSA-C diagnosis [14].

The utilization of dopamine transporter SPECT imaging (commonly known as DAT scan) was FDA approved for differentiating tremor with essential tremor (ET) from neurodegenerative parkinsonism in 2011. A normal DAT scan is characterized by maintaining the symmetric striatal signal (“comma shaped” appearance). While, an abnormal DAT scan is characterized by asymmetry of the signal, loss of activity in putamen, absent activity in both hemisphere or significantly reduced in one hemisphere. [18].

### 3.1 Approach for MSA-P diagnosis

Once clinical suspicion for MSA-P is established, the next step is differentiating the neurodegenerative parkinsonism from non-neurodegenerative parkinsonism. A DAT scan is a useful test in this context; it has over 90 % sensitivity and specificity in differentiating neurodegenerative from non-dopamine deficiency etiologies of parkinsonism [19]. Once neurodegenerative form of parkinsonism is established, the subsequent step is to differentiate synucleinopathies from tauopathies, followed by distinguishing MSA from Parkinson's disease (PD) and Lewy body dementia (Fig. 2).

#### 3.1.1 Synucleinopathy vs tauopathy

The most crucial step when diagnosing the MSA is to distinguish synucleinopathy from tauopathy. The synucleinopathy disorder includes PD, MSA and Lewy body dementia (DLB), while tauopathy causing parkinsonism include progressive supranuclear palsy (PSP) and corticobasal degeneration (CBD). Other tauopathy that are not related to parkinsonism include frontotemporal lobar degeneration (FTLD), Alzheimer’s disease and other rare tauopathy like argyrophilic grain disease. When differentiating between synucleinopathy and tauopathy, the most important consideration is to identify clinical signs that differentiate between the two entities. The roles of REM sleep behavior disorder (RBD) and anosmia are well-established in supporting the diagnosis of synucleinopathy, whereas these features are not commonly observed in tauopathy. Polysomnography-confirmed RBD has a positive predictive value of 98 % in distinguishing synucleinopathy from non-synucleinopathy disorders [20]. Signs such as downgaze supranuclear palsy or slowing of vertical saccades are suggestive of PSP. Similarly cortical sensory deficit, align-hand phenomena suggest the likelihood of CBD.

Pathology remains the most accurate test to differentiate synucleinopathy and tauopathy. Presence of alpha-synuclein in the CSF is a significant marker but it has limitation due to invasiveness of the procedure, Moreover, the detection of MSA among other synucleinopathies was not shown to be as sensitive with CSF synuclein seed amplification assay [21]. However, neurofilament light chain in CSF has demonstrated a sensitivity of 80 %-83 % and a specificity of 90 %-97 % in differentiating MSA from PD [20]. Recently, cutaneous alpha-phosphorylated synuclein markers is developed to identify synucleinopathies, even in early stages [22] (Fig. 1) with sensitivity of 95.5 % of detecting clinically-diagnosed synucleinopathy [23].

Once the differentiation of tauopathy and synucleinopathy is clear then the next question should investigate to clinically differentiating between various synucleinopathy, which can be a useful marker to differentiate these diseases further.

#### 3.1.2 MSA vs PD

Certain clinical characteristics can help distinguish between MSA and PD. Patients with PD exhibit a gradual progression of symptoms, whereas those with MSA experience a rapid progression. Autonomic dysfunction, which is a core clinical feature in the MDS-MSA criteria, can also present in PD; however, it typically emerges in the later stages of the disease. PD patients also demonstrate a sustained and optimal response to levodopa therapy, whereas MSA patients do not consistently respond optimally to levodopa treatment [16]. Anosmia is typically reported by PD patients, but unexplained anosmia is an exclusionary clinical criterion in the diagnosis of MSA [14]. Yoshita in 1998 looked at cardiac MIBG (Metaiodobenzylguanidine) scan in 25 PD patients, 15 SND patients, 14 PSP patients and 20 control subjects. They reported the mean value of H/M (heart/mediastinal) was significantly lower in PD patients compared to other patients and healthy subjects, postulating that MIBG can provide a useful diagnostic information in akinetic rigid Parkinson’s patients [24]. Cardiac MIBG scintigraphy is recognized as a supportive tool by the Movement Disorder Society (MDS) criteria for the diagnosis of clinically established and clinically probable MSA. However, various conditions, such as cardiac diseases, small fiber neuropathy, and the concomitant use of medications that affect the noradrenergic system, can influence the results of the MIBG test. Additionally, a mildly reduced MIBG uptake may be observed in early-stage MSA, while in early PD patients, the uptake might remain normal [14]. Cardiac MIBG scintigraphy with normal early and delayed heart-to-mediastinum ratios has shown a sensitivity of 83 % and 90 %-94 %, respectively, and a specificity of 89 % and 80 %-83 %, respectively [17]. Cardiovascular autonomic testing, such as Valsalva maneuver, heart rate variability during deep breathing, tilt table test and thermoregulatory test can provide a useful information to differentiate MSA and PD. The autonomic dysfunction in MSA is predominantly related with central pathology, while in PD, the autonomic dysfunction is predominantly related with peripheral involvement [25]. Supine plasma norepinephrine levels have been used for differentiation of MSA and PD. Reduced supine norepinephrine levels are reported with PD, whereas these levels are relatively preserved in MSA. However, it is important to note that this test exhibits low sensitivity but high specificity [14].

In addition to the aforementioned methods, elevated post-void residual (PVR) can also help differentiate between MSA and PD. Due to the high specificity of a PVR greater than 100 cc for diagnosing MSA, unexplained voiding difficulties with a PVR greater than 100 cc have been included as one of the autonomic dysfunctions in the criteria for clinically established MSA [14].

#### 3.1.3 MSA vs DLB

MSA and DLB are both classified as synucleinopathies. The hallmark clinical features with DLB are the presence of dementia, fluctuation in cognition, and visual hallucinations, while early onset dementia, early visual hallucinations, and fluctuation in cognition are exclusionary criteria for MSA [14], [26]. Beside clinical features, FDG-PET of brain and neuropsychological evaluation can assist with differentiating MSA and DLB.

Evaluation of predominant involvement of temporo-parieto-occipital hypometabolism can assist in differentiating DLB from non-DLB parkinsonism and Alzheimer’s disease [27]. Furthermore, cingulate island sign has shown to have good specificity for diagnosis of DLB [27]. Conversely, subcortical metabolic abnormalities are reported with MSA [25], [11]. A CSF neurofilament light chain level greater than 1400 pg/mL, measured by enzyme-linked immunosorbent assay, provides a sensitivity of 97 % and a specificity of 90 % for differentiating MSA from LBD [14]. Furthermore, neuropsychological evaluation can be a crucial tool in the diagnosis of DLB.

### 3.2 Approach for MSA-C diagnosis

MSA-C should be considered in patients with cerebellar ataxia accompanied by concurrent autonomic dysfunction. MRI brain imaging and DAT scans can be valuable diagnostic tools. If there is no or unclear evidence of autonomic dysfunction, or if there is no radiological evidence supporting an MSA-C etiology, other causes of cerebellar ataxia should be evaluated. A thorough history, including family history, personal history, and any history of substance abuse or potential medication use that could cause cerebellar ataxia, should be obtained. The sensitivity of neurogenic orthostatic hypotension in differentiating between early MSA-C and adult-onset sporadic cerebellar ataxia is about 32 %-56 %, with specificity of about 94 %-100 %. This changes to a sensitivity of 64 %-73 % and specificity of 100 % in advanced disease [28]. Additionally, a detailed but individualized laboratory assessment, cerebrospinal fluid (CSF) studies, and genetic testing should be considered (Fig. 3).

## 4 Conclusion

The diagnosis of MSA remains challenging despite two consensus MSA diagnostic criteria. Pathological confirmation relies on the presence of alpha-synuclein-positive GCIs and striatonigral/olivopontocerebellar degeneration. While MRI-brain is an essential diagnostic tool, it does not confirm the diagnosis of MSA. Recent 2022 MDS MSA criteria has shown 99 % specificity for clinically established MSA diagnosis but has a challenge of low sensitivity, while it demonstrated moderate sensitivity and specificity for clinically probable MSA cases [15]. In real world settings, application of these criteria can be challenging, especially in clinically complex cases; to address the complicated situations, we propose algorithms to assist the diagnosis of MSA. However, it is important to note that this should not replace clinical judgment or the use of well-established diagnostic criteria for MSA.

## CRediT authorship contribution statement

**Deepmala Nandanwar:** Writing – review & editing, Writing – original draft, Methodology, Investigation, Data curation, Conceptualization. **Daniel D. Truong:** Writing – review & editing, Supervision.

## Declaration of competing interest

The authors declare that they have no known competing financial interests or personal relationships that could have appeared to influence the work reported in this paper.
